# A novel nutritional index is associated with adverse outcomes in patients with acute heart failure: insights beyond traditional risk markers

**DOI:** 10.3389/fnut.2026.1962962

**Published:** 2026-10-26

**Authors:** Yue Cao, Bolan Zhang, Yi Wang, Yu Gao, Weiqi Luo, Yaoyu Qi, Lei Xia, Yi Zhong, Shuzhan Zheng, Qiang Ye

**Affiliations:** Department of Cardiology, The Affiliated Hospital of Southwest Medical University, Luzhou, Sichuan, China

**Keywords:** acute heart failure, all-cause mortality, heart failure rehospitalization, nutritional status, prognosis, triglyceride–total cholesterol–body weight index

## Abstract

**Background:**

Nutritional status is closely associated with clinical outcomes in patients with acute heart failure (AHF). However, the value of simple and comprehensive nutritional indicators in this population remains unclear. This study aimed to investigate the association between the triglyceride–total cholesterol–body weight index (TCBI) and adverse outcomes in patients with AHF.

**Methods:**

This single-center retrospective cohort study consecutively included patients with AHF admitted between April 2022 and April 2024. TCBI and conventional nutritional indices, including prognostic nutritional index (PNI), geriatric nutritional risk index (GNRI), and controlling nutritional status (CONUT), were calculated at admission. The primary outcome was a composite endpoint of heart failure rehospitalization or all-cause mortality within 1 year after discharge. Cox regression, restricted cubic spline analysis, threshold effect analysis, ROC analysis, subgroup analyses, and incremental model analyses were performed.

**Results:**

Among 1,898 patients with AHF, 557 experienced composite events during follow-up. After multivariable adjustment, higher TCBI was independently associated with a lower risk of the composite outcome (HR = 0.70, 95% CI: 0.60–0.81, *p* < 0.001). TCBI showed the highest discriminatory ability among nutritional indicators (AUC = 0.627). Restricted cubic spline and threshold effect analyses demonstrated a nonlinear association between TCBI and adverse outcomes. After adjustment for traditional cardiovascular risk factors and NT-proBNP, TCBI remained independently associated with outcomes. Compared with the base model including traditional cardiovascular risk factors and NT-proBNP, the addition of TCBI improved model performance, with the C-index increasing from 0.676 to 0.686 and the AUC from 0.703 to 0.716. The association between TCBI and outcomes remained consistent across HFrEF, HFmrEF, and HFpEF phenotypes.

**Conclusion:**

TCBI was independently associated with heart failure rehospitalization or all-cause mortality in patients with AHF and provided additional prognostic information beyond conventional nutritional indices and NT-proBNP.

## 1 Introduction

Acute heart failure (AHF) remains one of the leading causes of hospitalization and mortality among patients with cardiovascular diseases (1). Despite continuous advances in heart failure management strategies in recent years, patients with AHF continue to experience high rates of rehospitalization and mortality after discharge (2, 3). Therefore, identifying high-risk patients and implementing effective risk stratification strategies are essential for optimizing clinical management and improving patient outcomes.

In addition to traditional indicators of heart failure severity, the overall nutritional status of patients has increasingly been recognized as an important determinant associated with heart failure progression and clinical outcomes (4–6). Nutritional abnormalities are common among patients with heart failure and are associated with inflammatory activation, metabolic disturbances, muscle wasting, and disease progression (6–8). Previous studies have demonstrated that various nutritional assessment tools are associated with mortality and rehospitalization risk in patients with heart failure. Currently, traditional nutritional indices, including the prognostic nutritional index (PNI), geriatric nutritional risk index (GNRI), and controlling nutritional status (CONUT) score, have been widely used to evaluate nutritional status in patients with heart failure (9, 10). However, these indices generally rely on laboratory parameters such as serum albumin and may be influenced by inflammatory status, hepatic and renal dysfunction, and acute disease responses. Moreover, the strength of their associations with heart failure outcomes has not been entirely consistent across different studies. Therefore, identifying simple, readily available indicators that comprehensively reflect nutritional and metabolic status remains of important clinical interest.

Triglycerides, total cholesterol, and body weight are routinely available clinical parameters that reflect lipid metabolism and overall body composition characteristics. Based on these parameters, the triglyceride–total cholesterol–body weight index (TCBI) was developed as a novel composite indicator that integrates lipid metabolic status and body weight information (11, 12). Compared with traditional nutritional indices requiring albumin measurements, TCBI is simple to calculate and readily accessible in clinical practice. Previous studies have demonstrated associations between TCBI and clinical outcomes in various cardiovascular diseases, suggesting its potential value for cardiovascular risk assessment (13–16). However, the relationship between TCBI and clinical outcomes in patients with acute heart failure remains insufficiently investigated. Whether TCBI provides additional prognostic information beyond traditional nutritional indices and established heart failure biomarkers such as N-terminal pro-B-type natriuretic peptide (NT-proBNP) remains unclear. Furthermore, patients with different heart failure phenotypes, including heart failure with reduced ejection fraction (HFrEF), heart failure with mildly reduced ejection fraction (HFmrEF), and heart failure with preserved ejection fraction (HFpEF), exhibit differences in clinical characteristics and prognosis. Whether the association between TCBI and clinical outcomes is consistent across different heart failure phenotypes remains inadequately validated.

Therefore, this study aimed to evaluate the association between TCBI and the composite adverse outcome of heart failure rehospitalization or all-cause mortality in a cohort of hospitalized patients with acute heart failure and to compare its prognostic relevance with traditional nutritional indices, including PNI, GNRI, and CONUT. Furthermore, we performed stratified analyses to investigate the consistency of the association between TCBI and clinical outcomes across HFrEF, HFmrEF, and HFpEF patients, and evaluated whether TCBI provided additional prognostic information after adjustment for traditional clinical factors and NT-proBNP.

## 2 Methods

### 2.1 Study population

For analytical purposes in the current investigation, a single cohort was derived (Figure 1).

**Figure 1:**
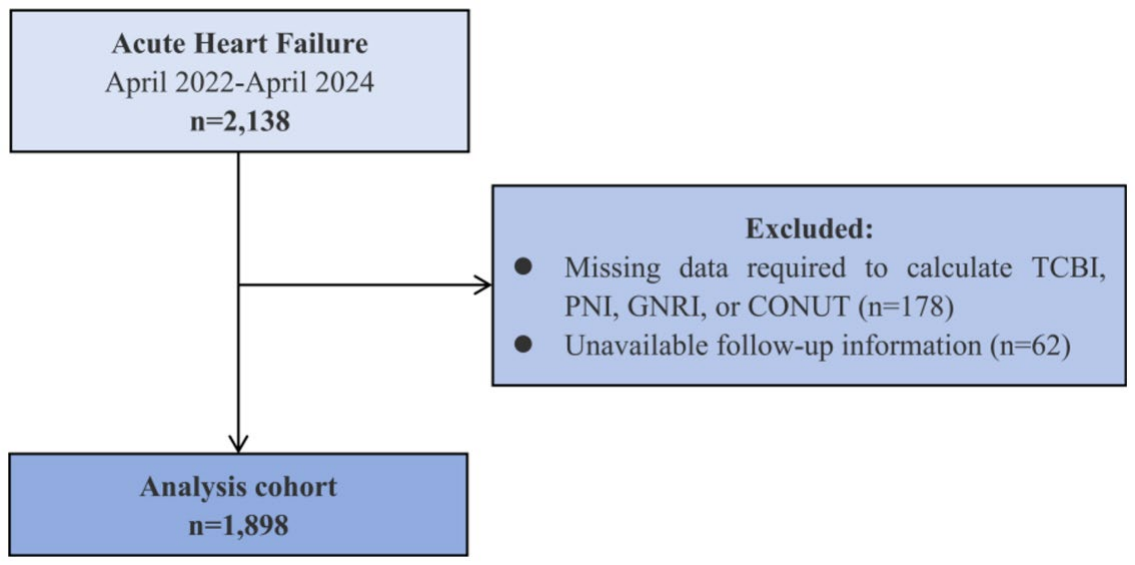
Flowchart of patient screening and selection for the final analysis cohort.

This study was a single-center retrospective cohort study designed to evaluate the association between nutritional indicators and clinical outcomes in patients with acute heart failure (AHF). Consecutive patients who were admitted to the Department of Cardiology at the Affiliated Hospital of Southwest Medical University between April 2022 and April 2024 and diagnosed with AHF were included.

The diagnosis of AHF was established based on clinical symptoms, physical signs, laboratory examinations, and imaging findings according to current guideline recommendations. Patients aged ≥18 years who met the diagnostic criteria for AHF were eligible for inclusion. During the study period, 2,138 patients were initially screened. According to the predefined eligibility criteria, 178 patients were excluded due to missing data required for calculating TCBI, PNI, GNRI, or CONUT, and 62 patients were excluded because of unavailable follow-up information. Finally, 1,898 patients were included in the final analysis cohort.

The study protocol was approved by the Clinical Trial Ethics Committee of the Affiliated Hospital of Southwest Medical University (Approval No. KY2026173). The study was conducted in accordance with the principles of the Declaration of Helsinki. Due to the retrospective design, the requirement for informed consent was waived by the ethics committee.

### 2.2 Clinical characteristics and data collection

Baseline clinical characteristics at admission were collected, including demographic characteristics, lifestyle factors, medical history, laboratory parameters, echocardiographic measurements, and medication use. Demographic characteristics included age, sex, height, weight, and body mass index (BMI). Lifestyle factors included smoking and drinking status. Medical history included hypertension, diabetes mellitus, coronary artery disease, stroke, and atrial fibrillation.

Medication information was obtained from electronic medical records and categorized according to therapeutic classes, including glucose-lowering therapy, lipid-lowering therapy, calcium channel blocker (CCB) therapy, anticoagulant therapy, antiplatelet therapy, diuretic therapy, and guideline-directed medical therapy (GDMT) for heart failure. Glucose-lowering therapy included insulin and metformin; lipid-lowering therapy included statins; CCB therapy included calcium channel blockers; anticoagulant therapy included oral anticoagulants; antiplatelet therapy included aspirin, indobufen, clopidogrel, ticagrelor, and cilostazol; and diuretic therapy included loop diuretics and thiazide diuretics. GDMT for heart failure included sodium–glucose cotransporter 2 inhibitors (SGLT2i), angiotensin-converting enzyme inhibitors/angiotensin receptor blockers/angiotensin receptor–neprilysin inhibitors (ACEI/ARB/ARNI), β-blockers, and mineralocorticoid receptor antagonists (MRAs).

Laboratory parameters included routine blood tests, biochemical measurements, lipid profiles, nutritional-related parameters, renal function indicators, and heart failure-related biomarkers. The main variables analyzed included triglycerides (TG), total cholesterol (TC), albumin (ALB), lymphocyte count, creatinine, urea, estimated glomerular filtration rate (eGFR), and N-terminal pro-B-type natriuretic peptide (NT-proBNP).

### 2.3 Assessment of TCBI

This study primarily evaluated the association between TCBI and clinical outcomes in patients with AHF. TCBI was calculated based on TG, TC, and body weight measured at admission (17):

$$\text{TCBI}=\frac{TG_{\left(\mathrm{mg}/\mathrm{dL}\right)}\times TC_{\left(\mathrm{mg}/\mathrm{dL}\right)}\times BW_{\left(\mathrm{kg}\right)}}{1000}$$

TCBI was natural logarithmically transformed, and the transformed values were used for baseline characteristic descriptions and all subsequent statistical analyses. Admission weight rather than documented dry weight was used. PNI, GNRI, and CONUT were calculated according to previously reported methods (18).

### 2.4 Covariates

Covariates included in the multivariable models were prespecified based on clinical relevance and previous evidence. The fully adjusted model incorporated demographic characteristics (age, sex, and BMI), lifestyle factors (smoking and drinking status), comorbidities (diabetes mellitus, hypertension, coronary artery disease, stroke, and atrial fibrillation), and medication use.

In an additional analysis, NT-proBNP was further added to the model to evaluate whether the association between TCBI and clinical outcomes remained independent after accounting for a conventional heart failure biomarker.

### 2.5 Study outcomes and follow-up

The primary outcome was a composite endpoint of heart failure rehospitalization or all-cause mortality within 1 year after discharge. All patients were followed for 1 year, and follow-up information was obtained from electronic medical records, outpatient records, and telephone interviews. The time to event was defined as the interval from discharge to the first occurrence of heart failure rehospitalization or all-cause mortality. Heart failure rehospitalization was defined as an unplanned readmission due to worsening heart failure. Routine check-ups, outpatient follow-up visits, planned admissions, and hospitalizations unrelated to heart failure were not counted as heart failure rehospitalization events.

### 2.6 Sample-size estimation

A supplementary sample-size calculation using the Hsieh–Lavori method assumed a two-sided α = 0.05, 80% power, HR = 0.80 per standard-deviation increase in TCBI, R^2^ = 0.20, and a 30% event rate. The required sample size was 730 patients after allowing for 10% loss to follow-up, below the 1,898 patients included in this study.

### 2.7 Statistical analysis

Continuous variables were presented as mean ± standard deviation or median (interquartile range) according to their distributions, while categorical variables were summarized as frequencies and percentages. Comparisons between groups were performed using the independent-samples *t* test or Mann–Whitney U test for continuous variables and the chi-square test or Fisher’s exact test for categorical variables.

Cox proportional hazards regression models were used to evaluate the associations between nutritional indicators and the composite outcome. Multivariable models were adjusted for potential confounders, including demographic characteristics, lifestyle factors, comorbidities, and medication use. NT-proBNP was additionally incorporated into the model to assess whether the association between TCBI and clinical outcomes remained independent after further adjustment for heart failure-related biomarkers.

Restricted cubic spline analyses were performed to examine the dose–response relationship between TCBI and the composite outcome, with further assessment of potential threshold effects. Receiver operating characteristic (ROC) curve analysis was used to compare the discriminatory ability of different nutritional indicators. The incremental value of TCBI was evaluated by comparing a baseline model with an extended model incorporating TCBI.

Subgroup analyses were conducted according to prespecified clinical characteristics, including heart failure phenotypes (HFrEF, HFmrEF, and HFpEF), smoking status, drinking status, diabetes mellitus, hypertension, coronary artery disease, history of stroke, and lipid-lowering therapy. Interaction tests were performed to assess the consistency of associations across subgroups. All statistical tests were two-sided, and *p* < 0.05 was considered statistically significant. All analyses were performed using R software (version 4.4.0).

## 3 Results

### 3.1 Baseline characteristics

A total of 1,898 patients with AHF were included in this study, including 1,341 patients without composite events and 557 patients who experienced composite events (heart failure rehospitalization or all-cause mortality) during the 1-year follow-up. Baseline characteristics are presented in Table 1. Compared with patients without events, those with composite events were older and had lower body weight and BMI. In addition, the proportions of diabetes mellitus, coronary artery disease, and atrial fibrillation were higher in the event group (all *p* < 0.05).

**Table 1:** Baseline characteristics of patients according to the occurrence of the composite endpoint.

Variable	No composite endpoint *N* = 1,341	Composite endpoint *N* = 557	*P* value
Age	69.00 [59.00; 77.00]	73.00 [66.00; 79.00]	<0.001
Sex, Male (%)	811 (60.48%)	334 (59.96%)	0.876
Height, cm	160.00 [155.00; 167.00]	160.00 [155.00; 167.00]	0.944
Weight, kg	60.00 [53.00; 70.00]	60.00 [50.00; 67.00]	<0.001
BMI, kg/m^2^	23.88 [21.01; 26.61]	22.77 [20.28; 25.25]	<0.001
Smoke status, *n* (%)	534 (39.82%)	207 (37.16%)	0.303
Drink status, *n* (%)	425 (31.69%)	160 (28.73%)	0.222
Heart rate, beats/min	83.00 [72.00; 97.00]	84.00 [71.00; 97.00]	0.405
SBP, mmHg	126.00 [111.00; 143.00]	125.00 [110.00; 142.00]	0.285
DBP, mmHg	79.00 [69.00; 91.00]	76.00 [67.00; 86.00]	<0.001
Hypertension, *n* (%)	734 (54.74%)	308 (55.30%)	0.863
Diabetes mellitus, *n* (%)	313 (23.34%)	181 (32.50%)	<0.001
CAD, *n* (%)	548 (40.87%)	332 (59.61%)	<0.001
Stroke, *n* (%)	172 (12.83%)	88 (15.80%)	0.101
Atrial fibrillation, *n* (%)	239 (17.82%)	154 (27.65%)	<0.001
Heart pacemaker, *n* (%)	30 (2.24%)	28 (5.03%)	0.002
Pulmonary embolism, *n* (%)	19 (1.42%)	9 (1.62%)	0.906
COPD, *n* (%)	126 (9.40%)	66 (11.85%)	0.126
Echocardiogram			
LA, mm	39.00 [33.00; 45.00]	39.00 [35.00; 45.00]	0.091
LVDd, mm	52.00 [46.00; 60.00]	52.00 [47.00; 60.00]	0.438
RV, mm	22.00 [20.00; 24.00]	22.00 [20.00; 24.00]	0.590
RA, mm	47.00 [42.00; 55.00]	48.00 [42.00; 57.00]	0.107
E/e′	24.29 [13.75; 42.50]	33.00 [17.00; 52.00]	<0.001
LVEF, %	50.00 [39.00; 60.00]	50.00 [40.00; 60.00]	0.939
Medication			
*Glucose-lowering therapy, n (%)*	246 (18.34%)	146 (26.21%)	<0.001
Insulin, *n* (%)	131 (9.77%)	86 (15.44%)	0.001
Metformin, *n* (%)	154 (11.48%)	86 (15.44%)	0.022
*Lipid-lowering therapy, n (%)*	365 (27.22%)	292 (52.42%)	<0.001
Statins, *n* (%)	365 (27.22%)	292 (52.42%)	<0.001
*Antihypertensive therapy, n (%)*	258 (19.24%)	135 (24.24%)	0.017
CCB, *n* (%)	258 (19.24%)	135 (24.24%)	0.017
*Anticoagulant therapy, n (%)*	154 (11.48%)	119 (21.36%)	<0.001
OAC, *n* (%)	154 (11.48%)	119 (21.36%)	<0.001
*Antiplatelet therapy, n (%)*	331 (24.68%)	243 (43.63%)	<0.001
Cilostazol, *n* (%)	44 (3.28%)	53 (9.52%)	<0.001
Clopidogrel/Ticagrelor, *n* (%)	257 (19.16%)	199 (35.73%)	<0.001
Aspirin/Indobufen, *n* (%)	237 (17.67%)	169 (30.34%)	<0.001
*Diuretic therapy, n (%)*	361 (26.92%)	286 (51.35%)	<0.001
Loop diuretics, *n* (%)	326 (24.31%)	277 (49.73%)	<0.001
Thiazide diuretics, *n* (%)	69 (5.15%)	38 (6.82%)	0.183
*GDMT, n (%)*	546 (40.72%)	359 (64.45%)	<0.001
SGLT2i, *n* (%)	160 (11.93%)	136 (24.42%)	<0.001
ACEI/ARB/ARNI, *n* (%)	357 (26.62%)	244 (43.81%)	<0.001
Beta-blockers, *n* (%)	375 (27.96%)	249 (44.70%)	<0.001
MRA, *n* (%)	341 (25.43%)	276 (49.55%)	<0.001
Blood test indicators			
FBG, mmol/L	5.95 [5.12; 7.40]	6.18 [5.15; 8.45]	0.053
NT-proBNP, pg/mL	3252.00 [1540.00; 7723.00]	5075.00 [2262.00; 11955.00]	<0.001
LDL-C, mmol/L	2.57 [2.01; 3.29]	2.27 [1.69; 2.87]	<0.001
HDL-C, mmol/L	1.14 [0.94; 1.41]	1.16 [0.93; 1.45]	0.605
TG, mmol/L	1.26 [0.98; 1.72]	1.09 [0.83; 1.53]	<0.001
TC, mmol/L	4.23 [3.52; 5.10]	3.90 [3.15; 4.74]	<0.001
APOA1, g/L	1.25 [1.07; 1.45]	1.24 [1.02; 1.42]	0.191
APOB, g/L	0.86 [0.69; 1.07]	0.76 [0.59; 0.92]	<0.001
HGB, g/L	130.00 [115.00; 144.00]	127.00 [111.00; 139.00]	<0.001
PLT, 10^9^/L	192.00 [150.00; 239.00]	176.00 [141.00; 225.00]	0.001
D-dimer, ug/ml	0.66 [0.35; 1.38]	0.73 [0.39; 1.46]	0.150
ALT, U/L	25.70 [16.00; 42.80]	22.10 [14.80; 40.40]	0.008
AST, U/L	29.60 [21.10; 49.80]	28.10 [20.50; 45.90]	0.143
TBIL, umol/L	13.80 [9.60; 20.10]	13.60 [9.90; 21.00]	0.571
UA, umol/L	400.30 [321.80; 505.30]	421.50 [328.50; 533.40]	0.055
UR, mmol/L	7.23 [5.56; 9.62]	8.32 [6.19; 11.62]	<0.001
Creatinine, umol/L	87.90 [71.80; 113.10]	96.50 [76.10; 141.10]	<0.001
eGFR, mL/min/1.73 m^2^	70.70 [51.20; 87.30]	61.70 [38.70; 81.30]	<0.001
CK-MB, ng/mL	2.84 [1.66; 5.25]	2.85 [1.79; 5.25]	0.961
hs-cTnT, ng/ml	0.04 [0.02; 0.18]	0.03 [0.02; 0.10]	0.583
Myo, ng/ml	52.01 [33.18; 100.30]	58.19 [38.76; 107.20]	0.007
ALB, g/L	40.00 [37.00; 42.50]	39.70 [36.40; 42.00]	0.068
Lymphocyte, 10^9^/L	1.23 [0.89; 1.70]	1.12 [0.79; 1.59]	0.002
Nutritional indicators			
TCBI (original values)	1113.66 [728.47; 1760.96]	805.46 [515.93; 1353.73]	<0.001
TCBI (log-transformed)	7.02 [6.59; 7.47]	6.69 [6.25; 7.21]	<0.001
PNI	46.35 [42.35; 50.20]	45.30 [41.75; 49.30]	0.010
GNRI	100.22 [94.23; 104.24]	98.58 [92.92; 102.75]	0.001
CONUT	3.00 [1.00; 4.00]	3.00 [2.00; 4.00]	<0.001
EF group			0.464
HFrEF	353 (26.32%)	134 (24.06%)	
HFmrEF	311 (23.19%)	141 (25.31%)	
HFpEF	677 (50.48%)	282 (50.63%)	

Data are presented as mean (standard deviation), median (interquartile range), or number (percentage). BMI, body mass index; SBP, systolic blood pressure; DBP, diastolic blood pressure; CAD, coronary artery disease; COPD, chronic obstructive pulmonary disease; LA, left atrial dimension; LVDd, left ventricular end-diastolic dimension; RV, right ventricular diameter; RA, right atrial dimension; LVEF, left ventricular ejection fraction; CCB, calcium channel blocker; OAC, oral anticoagulant; GDMT, guideline-directed medical therapy; SGLT2i, sodium–glucose cotransporter 2 inhibitor; ACEI, angiotensin-converting enzyme inhibitor; ARB, angiotensin receptor blocker; ARNI, angiotensin receptor–neprilysin inhibitor; MRA, mineralocorticoid receptor antagonist; FBG, fasting blood glucose; NT-proBNP, N-terminal pro-B-type natriuretic peptide; LDL-C, low-density lipoprotein cholesterol; HDL-C, high-density lipoprotein cholesterol; TG, triglyceride; TC, total cholesterol; APOA1, apolipoprotein A1; APOB, apolipoprotein B; HGB, hemoglobin; PLT, platelet count; ALT, alanine aminotransferase; AST, aspartate aminotransferase; TBIL, total bilirubin; UA, uric acid; UR, urea; eGFR, estimated glomerular filtration rate; CK-MB, creatine kinase MB; hs-cTnT, high-sensitivity cardiac troponin T; Myo, myoglobin; ALB, albumin; HFrEF, heart failure with reduced ejection fraction; HFmrEF, heart failure with mildly reduced ejection fraction; HFpEF, heart failure with preserved ejection fraction.

Regarding laboratory parameters, patients with composite events had higher levels of NT-proBNP, urea, and creatinine, along with lower eGFR levels (all *p* < 0.05). Echocardiographic assessment showed no significant differences in LVEF or heart failure phenotype distribution between groups, whereas E/e′ differed significantly between the two groups.

Regarding medication use, patients in the event group had higher proportions of glucose-lowering therapy, lipid-lowering therapy, anticoagulant therapy, antiplatelet therapy, diuretic therapy, and heart failure-related guideline-directed medical therapy (all *p* < 0.05).

For nutritional indicators, the event group had significantly lower TCBI values compared with the non-event group (6.69 [6.25–7.21] vs. 7.02 [6.59–7.47], *p* < 0.001). Furthermore, the event group showed lower PNI and GNRI values and higher CONUT scores (all *p* < 0.05).

### 3.2 Association between nutritional indicators and adverse outcomes

Cox proportional hazards regression models were performed to further evaluate the associations between nutritional indicators and composite outcomes (Table 2). In continuous variable analyses, TCBI showed a significant inverse association with the composite outcome after multivariable adjustment. Each one-unit increase in TCBI was associated with a lower risk of heart failure rehospitalization or all-cause mortality (HR = 0.70, 95% CI: 0.60–0.81, *p* < 0.001).

**Table 2:** Associations between nutritional indices and the composite outcome in patients with acute heart failure.

Variable	Model 1		Model 2		Model 3	
	HR (95% CI)	*P* value	HR (95% CI)	*P* value	HR (95% CI)	*P* value
TCBI	0.55 (0.49, 0.63)	<0.001	0.61 (0.53, 0.71)	<0.001	0.70 (0.60, 0.81)	<0.001
CONUT	1.12 (1.08, 1.17)	<0.001	1.09 (1.04, 1.13)	<0.001	1.05 (1.01, 1.10)	0.018
PNI	0.99 (0.98, 1.00)	0.205	1.00 (0.99, 1.01)	0.870	1.00 (0.99, 1.01)	0.939
GNRI	0.99 (0.98, 1.00)	0.064	1.01 (1.00, 1.02)	0.219	1.00 (0.99, 1.01)	0.753

HR, Hazard Ratio; CI, Confidence Interval.

Model 1: Non-adjusted.

Model 2: Adjusted for age, sex, BMI, smoking and drinking.

Model 3: Further adjusted for diabetes mellitus, hypertension, CAD, Stroke, atrial fibrillation, glucose-lowering therapy, lipid-lowering therapy, antihypertensive therapy, anticoagulant therapy, diuretic therapy, and GDMT based on model 2.

Among traditional nutritional indicators, CONUT score was positively associated with the composite outcome. In the fully adjusted model, each one-point increase in CONUT score was associated with an increased risk of composite events (HR = 1.05, 95% CI: 1.01–1.10, *p* = 0.018). In contrast, neither PNI nor GNRI showed significant associations with the composite outcome after full adjustment (both *p* > 0.05).

### 3.3 Dose–response relationship between TCBI and adverse outcomes

Restricted cubic spline (RCS) analysis was performed to evaluate the potential dose–response relationship between TCBI and the composite adverse outcome (Figure 2). A significant nonlinear association was observed between TCBI and the risk of composite events (P for overall <0.001; P for nonlinearity = 0.001). The risk of composite events gradually decreased with increasing TCBI levels.

**Figure 2:**
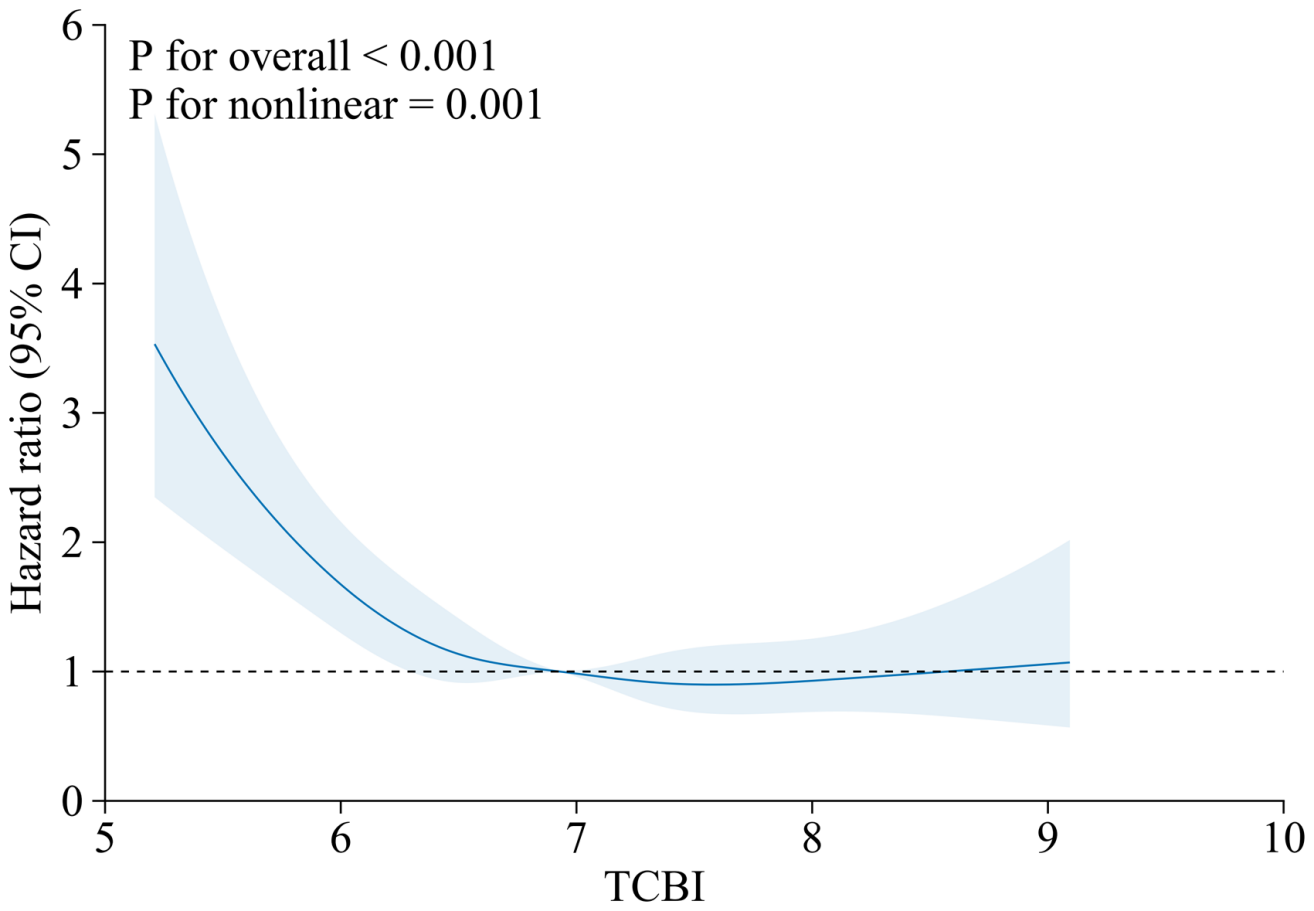
Restricted cubic spline analysis of the association between TCBI and the composite endpoint in acute heart failure.

### 3.4 Threshold effect analysis of TCBI and adverse outcomes

Threshold effect analysis showed a significant threshold effect between TCBI and the composite adverse outcome (P for likelihood ratio test <0.001) (Table 3). In the multivariable linear model, TCBI was significantly associated with the composite outcome (HR = 0.70, 95% CI: 0.60–0.81, *p* < 0.001). The two-piecewise regression model identified an inflection point of 6.67 for TCBI. When TCBI was below 6.67, higher TCBI levels were associated with a lower risk of composite outcomes (HR = 0.43, 95% CI: 0.33–0.57, *p* < 0.001). However, when TCBI was ≥6.67, no significant association was observed between TCBI and the composite outcome (HR = 0.97, 95% CI: 0.78–1.20, *p* = 0.746).

**Table 3:** Threshold effect analysis of TCBI on the composite endpoint using a two-piecewise regression model.

Outcome	HR (95%CI)	*P* value
Model 1 Fitting model by standard regression	0.70 (0.60, 0.81)	<0.001
Model 2 Fitting model by two-piecewise regression		
*Inflection point*		
TCBI <6.67	0.43 (0.33, 0.57)	<0.001
TCBI ≥6.67	0.97 (0.78, 1.20)	0.746
P for the likelihood test		<0.001

### 3.5 Comparison of prognostic performance among nutritional indicators

Receiver operating characteristic (ROC) curve analysis was performed to compare the discriminatory ability of different nutritional indicators for the 1-year composite adverse outcome (Figure 3). TCBI showed the highest area under the curve (AUC = 0.627), followed by CONUT (AUC = 0.589), GNRI (AUC = 0.549), and PNI (AUC = 0.538).

**Figure 3:**
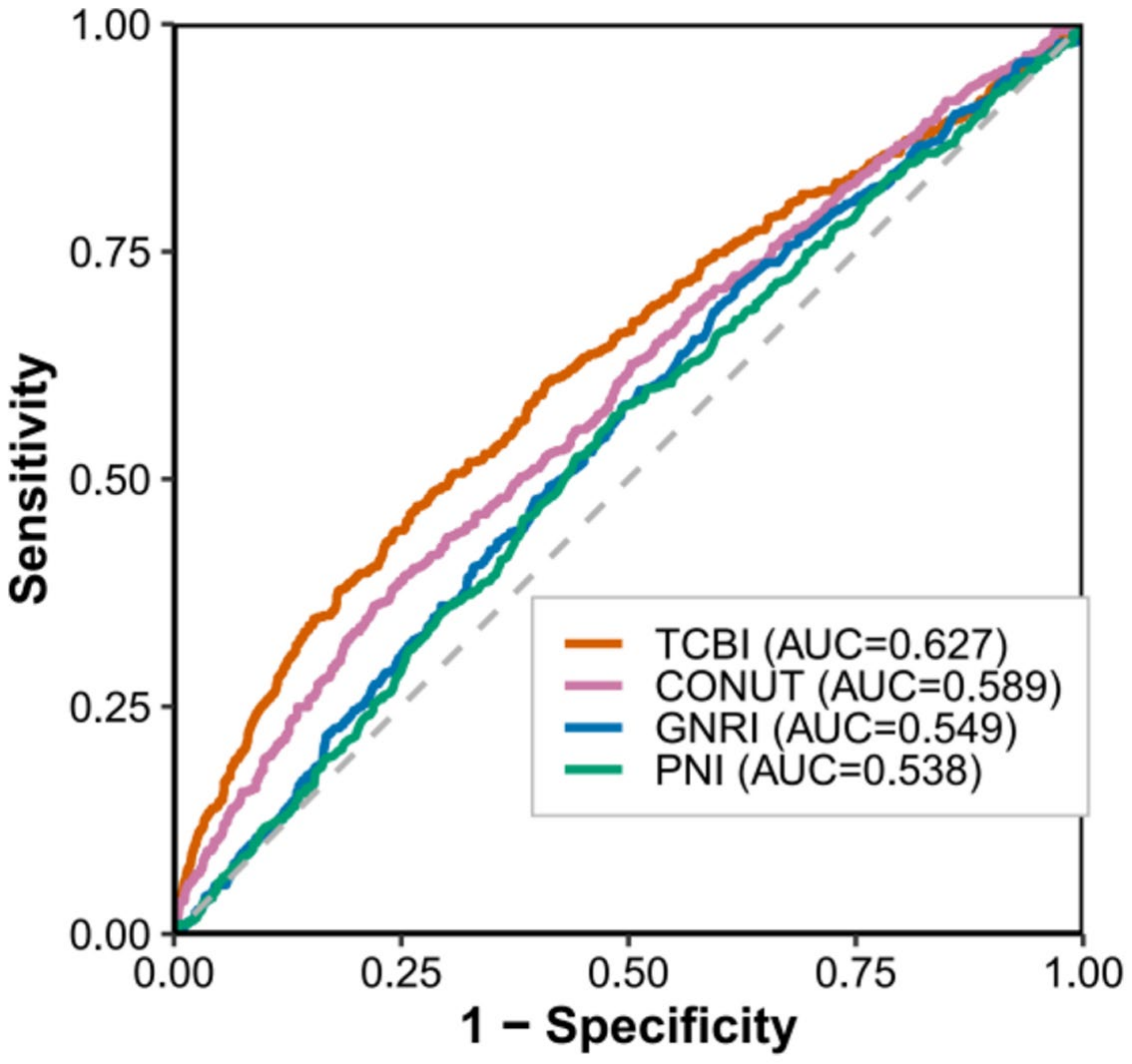
Receiver operating characteristic curves of TCBI, CONUT, GNRI, PNI.

### 3.6 Incremental prognostic value of TCBI beyond NT-proBNP

The incremental value of TCBI beyond NT-proBNP was assessed by comparing a base model incorporating conventional clinical factors and NT-proBNP with an extended model that additionally included TCBI. After adjustment for conventional clinical factors and NT-proBNP, TCBI remained independently associated with the 1-year composite adverse outcome (HR = 0.70, 95% CI: 0.61–0.82, *p* < 0.001). Compared with the base model, the addition of TCBI significantly improved model fit (likelihood ratio test: *p* < 0.001). Furthermore, incorporation of TCBI increased the C-index from 0.676 to 0.686 and the AUC from 0.703 to 0.716 (Table 4).

**Table 4:** Model performance before and after the addition of TCBI.

Model	Variables included	C-index	AUC
Model 1	Clinical covariates and NT-proBNP	0.676	0.703
Model 2	Clinical covariates, NT-proBNP, and TCBI	0.686	0.716

### 3.7 Comparison of TCBI with its lipid components

After adjustment for all covariates, Cox models incorporating either TCBI or TG and TC were compared. The C-index was 0.683 for the TCBI model and 0.680 for the model incorporating TG and TC, with a difference of 0.0031 (95% CI: 0.0004–0.0059; *p* = 0.026).

### 3.8 Subgroup analysis

The association between TCBI and the composite adverse outcome remained consistent across prespecified clinical subgroups (Table 5). Stratified analyses according to heart failure phenotype demonstrated that higher TCBI was associated with a lower risk of composite outcomes in patients with HFrEF, HFmrEF, and HFpEF. The corresponding HRs were 0.55 (95% CI: 0.39–0.78, *p* < 0.001), 0.65 (95% CI: 0.48–0.87, *p* = 0.004), and 0.77 (95% CI: 0.63–0.94, *p* = 0.012), respectively.

**Table 5:** Subgroup analyses of the association between TCBI and the composite outcome.

Character	HR (95%CI)	*P* value	P for interaction
EF group			0.183
HFrEF	0.55 (0.39, 0.78)	<0.001	
HFmrEF	0.65 (0.48, 0.87)	0.004	
HFpEF	0.77 (0.63, 0.94)	0.012	
Smoking			0.228
No	0.65 (0.53, 0.79)	<0.001	
Yes	0.78 (0.62, 0.996)	0.046	
Drinking			0.370
No	0.66 (0.55, 0.79)	<0.001	
Yes	0.81 (0.62, 1.07)	0.140	
Diabetes mellitus			0.001
No	0.59 (0.49, 0.71)	<0.001	
Yes	0.97 (0.76, 1.23)	0.779	
Hypertension			0.472
No	0.73 (0.58, 0.93)	0.009	
Yes	0.68 (0.56, 0.82)	<0.001	
CAD			< 0.001
No	0.51 (0.40, 0.66)	<0.001	
Yes	0.85 (0.71, 1.03)	0.092	
Stroke			0.674
No	0.70 (0.60, 0.83)	<0.001	
Yes	0.71 (0.49, 1.03)	0.075	
Lipid-lowering therapy			0.153
No	0.61 (0.49, 0.76)	<0.001	
Yes	0.79 (0.65, 0.97)	0.023	

Across other clinical subgroups, no significant interactions were observed for smoking status, drinking status, hypertension, or history of stroke (all P for interaction >0.05). Significant interactions were observed for diabetes mellitus and coronary artery disease (P for interaction = 0.001 and <0.001, respectively), with stronger associations observed among patients without diabetes mellitus and those without coronary artery disease. The inverse association was also observed in patients without lipid-lowering therapy (HR = 0.61, 95% CI: 0.49–0.76, *p* < 0.001) and those receiving lipid-lowering therapy (HR = 0.79, 95% CI: 0.65–0.97, *p* = 0.023), with no significant interaction (P for interaction = 0.153).

## 4 Discussion

In this study, we investigated the association between nutritional indicators and the composite outcome of heart failure rehospitalization or all-cause mortality in a cohort of patients with acute heart failure. Several important findings were identified. First, TCBI showed a significant inverse association with adverse clinical outcomes, with higher TCBI levels associated with a lower risk of composite events after adjustment for established clinical factors. Second, TCBI demonstrated superior discriminatory ability compared with conventional nutritional indices, including PNI, GNRI, and CONUT. Third, spline and threshold analyses revealed a nonlinear relationship between TCBI and adverse outcomes, suggesting a potential threshold pattern in this association. Fourth, the association between TCBI and outcomes remained significant after additional adjustment for NT-proBNP, and incorporation of TCBI improved model performance beyond conventional clinical factors and established heart failure biomarkers. Furthermore, the consistent association observed across HFrEF, HFmrEF, and HFpEF phenotypes supports the potential clinical relevance of TCBI across different heart failure subtypes.

In this single-center cohort of patients with acute heart failure, higher TCBI levels were independently associated with a lower risk of the composite outcome of heart failure rehospitalization or all-cause mortality. TCBI also showed better discrimination than the conventional nutritional indices PNI, GNRI, and CONUT. Spline and threshold analyses suggested a nonlinear association between TCBI and the composite outcome. This association remained significant after further adjustment for NT-proBNP, and adding TCBI improved model fit. Similar associations were observed across patients with HFrEF, HFmrEF, and HFpEF. These findings suggest that TCBI may provide a simple and readily available measure for assessing adverse outcome risk in patients with acute heart failure.

Patients with composite events were older and had a greater burden of diabetes mellitus, coronary artery disease, and atrial fibrillation, together with higher NT-proBNP levels and poorer renal function. These differences indicate a higher baseline clinical risk in the event group, which should be considered when interpreting the association between lower TCBI and adverse outcomes. The higher prevalence of medication use may reflect greater comorbidity burden and differences in treatment indications; it should not be interpreted as evidence that these treatments caused adverse outcomes.

Nutritional abnormalities are common among patients with heart failure and have been closely linked to disease progression and adverse clinical outcomes. Multiple factors, including chronic inflammatory activation, metabolic disturbances, reduced appetite, gastrointestinal dysfunction, and alterations in volume status, may contribute to inadequate nutritional intake and increased catabolic activity in this population (19, 20). Previous studies have demonstrated that various nutritional assessment tools are associated with mortality and rehospitalization risk among patients with heart failure (10, 21). However, commonly used nutritional indices largely depend on parameters such as serum albumin, lymphocyte count, and cholesterol levels, which may be influenced by inflammatory responses, hepatic dysfunction, and acute disease conditions (22). Particularly in patients with acute heart failure, the presence of systemic stress responses during hospitalization may limit the ability of individual nutritional markers to fully capture overall metabolic and nutritional status. Therefore, the identification of simple and comprehensive nutritional indicators remains clinically relevant.

TCBI is a composite index incorporating triglyceride, total cholesterol, and body weight, allowing simultaneous assessment of lipid metabolism and body composition without requiring serum albumin measurement (23). In the present study, TCBI showed a significant inverse association with adverse outcomes in patients with acute heart failure, with a stronger association compared with conventional nutritional indices. This finding may be explained by the multidimensional information captured by the components of TCBI. Decreased body weight may reflect disease-related wasting, whereas alterations in lipid levels may represent changes in energy reserve, metabolic adaptation, and systemic disease status (24, 25). By integrating these parameters, TCBI may provide a broader reflection of nutritional and metabolic conditions than individual indicators alone. In contrast, PNI, GNRI, and CONUT rely heavily on albumin-related measurements, and albumin levels can be affected by inflammation, fluid status, and other acute clinical conditions, potentially limiting their stability in patients with acute heart failure.

The relationship between TCBI and clinical outcomes was further explored using restricted cubic spline and threshold analyses. The observed nonlinear association indicated that the prognostic relevance of TCBI may vary across different ranges of values. Specifically, the association between lower TCBI levels and adverse outcomes was more pronounced below the identified threshold, whereas the association became attenuated at higher TCBI levels. These findings provide additional insight beyond simple categorical comparisons and suggest that reduced TCBI may help identify a subgroup of patients with acute heart failure who are at increased clinical risk.

NT-proBNP is one of the most widely used biomarkers in heart failure and primarily reflects ventricular wall stress and hemodynamic burden (26). However, clinical outcomes in patients with heart failure are determined not only by cardiac dysfunction but also by systemic conditions, including nutritional and metabolic status (20). In this study, TCBI remained independently associated with adverse outcomes after adjustment for NT-proBNP and further improved model performance when added to a model containing conventional clinical factors and NT-proBNP. These findings indicate that TCBI and NT-proBNP may capture different aspects of disease status: NT-proBNP reflects cardiac loading conditions, whereas TCBI may provide complementary information regarding nutritional and metabolic characteristics. Therefore, TCBI may serve as a supplementary marker alongside established heart failure biomarkers.

The association between TCBI and clinical outcomes was also consistent across different heart failure phenotypes, including HFrEF, HFmrEF, and HFpEF. Previous studies have demonstrated substantial differences among these phenotypes regarding underlying mechanisms, treatment strategies, and prognosis (26). Nevertheless, nutritional impairment may represent an important factor influencing disease progression across the spectrum of heart failure (27, 28). The consistent findings across phenotypes suggest that the clinical relevance of TCBI may not be restricted to a specific heart failure subtype but may extend to patients with diverse forms of heart failure.

The findings of this study may have several clinical implications. First, TCBI is derived from routinely available clinical parameters and does not require additional specialized testing, supporting its potential applicability in routine clinical settings. Second, compared with conventional nutritional indices, TCBI avoids dependence on albumin measurements and provides a simple approach for evaluating nutritional and metabolic status. Furthermore, beyond demonstrating an independent association with outcomes, this study also examined the incremental value of TCBI beyond NT-proBNP, providing additional evidence regarding its potential role in the comprehensive assessment of patients with acute heart failure.

Several limitations should be acknowledged. First, this was a single-center retrospective cohort study, and residual confounding and selection bias cannot be completely excluded. Future validation in multicenter prospective cohorts is warranted. Second, TCBI was assessed only at admission, and longitudinal changes in nutritional status during follow-up were not available. Future studies should investigate whether dynamic changes in TCBI provide additional prognostic information. Third, although comprehensive clinical variables were included in the adjustment models, unmeasured factors, such as dietary patterns, physical activity, and muscle mass, may have contributed to residual confounding. Fourth, admission weight rather than dry weight was used, and reliable congestion data were unavailable; therefore, fluid retention may have influenced TCBI. Finally, although statin use was adjusted for, residual effects of lipid-lowering treatment cannot be completely excluded.

## 5 Conclusion

TCBI was independently associated with the composite outcome of heart failure rehospitalization or all-cause mortality in patients with acute heart failure and showed better discrimination than conventional nutritional indices. This association remained significant after adjustment for NT-proBNP and was consistent across heart failure phenotypes. TCBI may therefore provide a simple, readily available nutritional-metabolic measure that complements clinical risk assessment in acute heart failure.

## Data Availability

The raw data supporting the conclusions of this article will be made available by the authors, without undue reservation.

## Glossary

AHF: acute heart failure
TCBI: triglyceride–total cholesterol–body weight index
PNI: prognostic nutritional index
GNRI: geriatric nutritional risk index
CONUT: controlling nutritional status
NT-proBNP: N-terminal pro-B-type natriuretic peptide
HFrEF: heart failure with reduced ejection fraction
HFmrEF: heart failure with mildly reduced ejection fraction
HFpEF: heart failure with preserved ejection fraction
LVEF: left ventricular ejection fraction
BMI: body mass index
eGFR: estimated glomerular filtration rate
ROC: receiver operating characteristic
AUC: area under the curve
C-index: concordance index
RCS: restricted cubic spline
HR: hazard ratio
CI: confidence interval
GDMT: guideline-directed medical therapy.
GNRI: geriatric nutritional risk index
PNI: prognostic nutritional index
CONUT: controlling nutritional status
